# Targeting Cytokines, Pathogen-Associated Molecular Patterns, and Damage-Associated Molecular Patterns in Sepsis via Blood Purification

**DOI:** 10.3390/ijms22168882

**Published:** 2021-08-18

**Authors:** Kazuhiro Moriyama, Osamu Nishida

**Affiliations:** 1 Laboratory for Immune Response and Regulatory Medicine, Fujita Health University School of Medicine, Toyoake 470-1192, Japan; 2 Department of Anesthesiology and Critical Care Medicine, Fujita Health University School of Medicine, Toyoake 470-1192, Japan; nishida@fujita-hu.ac.jp

**Keywords:** adsorption, blood purification, cytokine, hemofiltration, lipopolysaccharide, DAMPs, sepsis

## Abstract

Sepsis is characterized by a dysregulated immune response to infections that causes life-threatening organ dysfunction and even death. When infections occur, bacterial cell wall components (endotoxin or lipopolysaccharide), known as pathogen-associated molecular patterns, bind to pattern recognition receptors, such as toll-like receptors, to initiate an inflammatory response for pathogen elimination. However, strong activation of the immune system leads to cellular dysfunction and ultimately organ failure. Damage-associated molecular patterns (DAMPs), which are released by injured host cells, are well-recognized triggers that result in the elevation of inflammatory cytokine levels. A cytokine storm is thus amplified and sustained in this vicious cycle. Interestingly, during sepsis, neutrophils transition from powerful antimicrobial protectors into dangerous mediators of tissue injury and organ dysfunction. Thus, the concept of blood purification has evolved to include inflammatory cells and mediators. In this review, we summarize recent advances in knowledge regarding the role of lipopolysaccharides, cytokines, DAMPs, and neutrophils in the pathogenesis of sepsis. Additionally, we discuss the potential of blood purification, especially the adsorption technology, for removing immune cells and molecular mediators, thereby serving as a therapeutic strategy against sepsis. Finally, we describe the concept of our immune-modulating blood purification system.

## 1. Introduction

Sepsis and septic shock are severe inflammatory conditions that are associated with high morbidity and mortality rates [1]. The innate immune system is the first line of defense against bacterial infections [2]. The host defense system can recognize molecular components of invading pathogens, called pathogen-associated molecular patterns (PAMPs), with specialized receptors known as pattern recognition receptors (PRRs) [3,4]. Lipopolysaccharide (LPS), commonly known as a bacterial endotoxin, is a well-known PAMP that is a component of the outer membrane of gram-negative bacteria. Early studies have demonstrated that the stimulation of Toll-like receptor 4 (TLR4) by LPS induces the release of critical proinflammatory cytokines. As LPS plays a central role in triggering septic shock, new anti-endotoxin agents or TLR4 receptor antagonists that can block and prevent such aggravated responses serve as promising treatments [5]. However, it was recently discovered that LPS not only acts extracellularly through TLR4, but also intracellularly through the activation of caspases/inflammasomes to induce inflammatory responses and cell death [6,7]. In addition, the mechanism of endotoxin internalization into cells from the blood has recently been clarified [8], and the significance of endotoxins in the blood has been of interest [9,10].

Sepsis is defined as a life-threatening organ dysfunction caused by a dysregulated host response to infection [11]. This definition appropriately focuses on the deleterious and dysregulated host response as the principal pathophysiological event in sepsis. However, this definition implies that while infection might be the initiating factor causing sepsis, pathogens play little or no role in the generation of the potentially fatal sequence of events that occur in septic patients [12]. Subsequent cellular injury may lead to the release of various cytokines and endogenous molecules by injured cells, called danger-associated molecular patterns (DAMPs) or alarmins, leading to deterioration in a vicious cycle by further stimulation of PRRs [13,14]. This dysregulated immune response to infection is associated with a failure to return to homeostasis and harms the host, resulting in cellular dysfunction and, ultimately, organ failure [15]. Although studies on the pathogenesis of sepsis have rapidly increased, this complex syndrome is not yet fully understood. Moreover, our increased understanding of the pathophysiological mechanisms of sepsis has failed to improve health outcomes [16]. Nonetheless, based on the recently proposed pathophysiology of sepsis, the approach of targeting cytokines and DAMPs is more attractive than before [17,18].

Besides adequate source control and appropriate antibiotics, no specific therapy exists for sepsis. Consequently, much effort has been focused on alternative treatment strategies to improve outcomes. The application of blood purification has been suggested; however, this approach, which includes high-volume hemofiltration or high cut-off dialysis, has had little success to date [19]. More recently, the use of adsorption technologies has attracted significant attention [20]. Adsorption is more efficient at higher concentrations of the target substance, particularly inflammatory mediators from the bloodstream [21]. In this review, we examine the evolving concept of sepsis and discuss new and potential blood purification therapies.

## 2. LPS Recognition Systems

LPS has been extensively investigated and acknowledged as one of the key triggers of lethal shock and is one of the primary drivers of cytokine storm [22]. LPS is thought to form a complex with LPS-binding protein (LBP) and CD14 and bind to TLR4 on the surface of macrophages, ultimately triggering an excessive immune response and causing multi-organ dysfunction due to a cytokine storm [23,24]. LPS is thus considered an important target for elimination as an upstream trigger of sepsis [25]. TLR-targeted therapies are potent treatments for the prevention and intervention of infectious diseases, notably sepsis, during this period [5]. However, a clinical trial of inhibitors of TLR4 signaling (ACCESS study reported in 2013 [26]) reported negative results, casting a shadow on the importance of LPS-TLR4-targeted therapies in sepsis [27].

Alternative intracellular LPS-sensing pathways besides TLR4 have also been discovered [6,7]. Kayagaki et al. reported that the extracellular effects on TLRs other than LPS (poly(I:C)-stimulated signals from TLR3 in mouse experiments) resulted in cell activation similar to that of LPS/TLR4 signaling. However, in mice where the caspase-11 gene was knocked out, LPS-TLR4 signaling did not cause cell lysis or death [6]. Thus, caspase-11 was identified to be essential for the LPS-induced novel programmed cell death, pyroptosis [6,7,28,29]. With this discovery, pyroptosis was redefined as gasdermin-mediated programmed necrosis [30].

**Figure 1:**
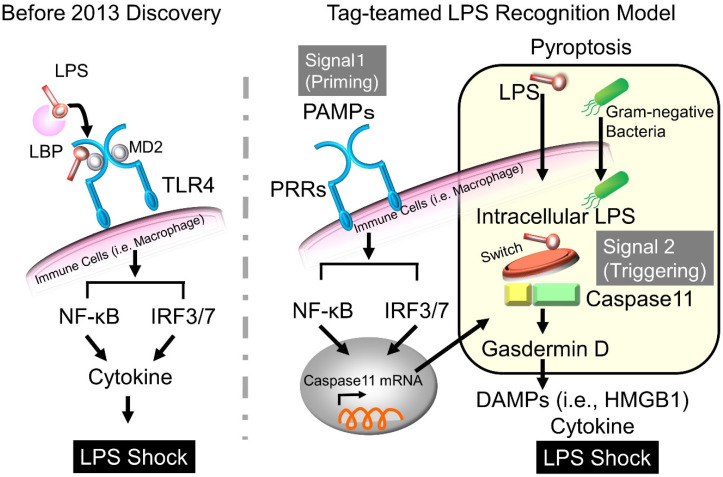
Tag-teamed LPS recognition model. Induction of LPS-mediated lethal sepsis proceeds in a two-step approach. The first signal is initiated by multiple TLRs in response to PAMPs (signal 1: priming) while the second signal in response to intracellular LPS triggers caspase-11 activation (signal 2: triggering), leading to lethal septic shock. Pyroptosis, leading to the passive release of intracellular inflammatory molecules such as HMGB1, is responsible for the lethal septic response. DAMPs, damage-associated molecular patterns; HMGB1, high mobility group box-1 protein; IRF, interferon regulatory factor; LBP, LPS-binding protein; LPS, lipopolysaccharide; NF-κB, nuclear factor-kappa B; PAMPs, pathogen-associated molecular patterns; PRRs, pattern-recognition receptors; MD2: not an abbreviation.

Pyroptosis requires two sequential signals: (i) priming and (ii) triggering; accordingly, it is referred to as a one-two-punch model for non-canonical inflammasome activation [31]. The first signal, priming, is initiated by multiple TLRs in response to bacterial PAMPs. Multiple TLRs induce the expression of the inflammasome components, NLRP3, pro-caspase-11, and pro-interleukin (IL)-1β, which justifies why TLR4 antagonists have not been found to work well. The second signal is intracellular LPS recognition that triggers caspase-11 (caspase-11 in mice and caspase-4/5 in humans). Sensing of LPS within the cell can trigger caspase-11 activation, leading to pyroptotic cell death. Pyroptosis features violent plasma-membrane damage leading to the passive release of intracellular inflammatory DAMPs, such as high mobility group box l protein (HMGB1) [28,31] (Figure 1). Cytoplasmic LPS sensing by caspase-11 plays a protective role during bacterial infection [32,33]. However, it can also cause tissue damage, disseminated intravascular coagulation syndrome, organ failure, and death [34,35]. This novel intracellular LPS sensing pathway provides a new paradigm for LPS-triggered endotoxemia development, with potential implications for the pharmacological treatment of sepsis and septic shock [10,36].

## 3. LPS-Induced Vascular Endothelial Injury

Although LPS-induced pyroptosis was previously defined as the death of mainly immune cells, such as macrophages, in 2017, Cheng et al. reported that LPS was directly taken up by pulmonary vascular endothelial cells to induce pyroptosis, resulting in vascular hyperpermeability pulmonary edema [8]. In their research, pyroptosis was induced and intracellular enzymes were released when LPS was introduced into cultured human vascular cells from foreign cells using transfection reagents; however, pyroptosis was not induced when LPS was applied using foreign cells. Based on this new finding, LPS in the blood is taken up by vascular endothelial cells and causes lung injury by pyrotosis [37]. LPS-induced pyroptosis has also been confirmed in organs besides the lungs, such as in tubular epithelial cells [38], cardiomyocytes [39], graft-versus-host disease [40], and neutrophils [41]. The gut, which is considered an important source of LPS in the bloodstream, has a neutralization mechanism for LPS [42], and the disruption of the intestinal barrier in sepsis plays a key role by promoting the presence of LPS in the bloodstream. As LPS directly causes cell injury, the significance of LPS removal by blood purification has escalated [36,43].

**Figure 2:**
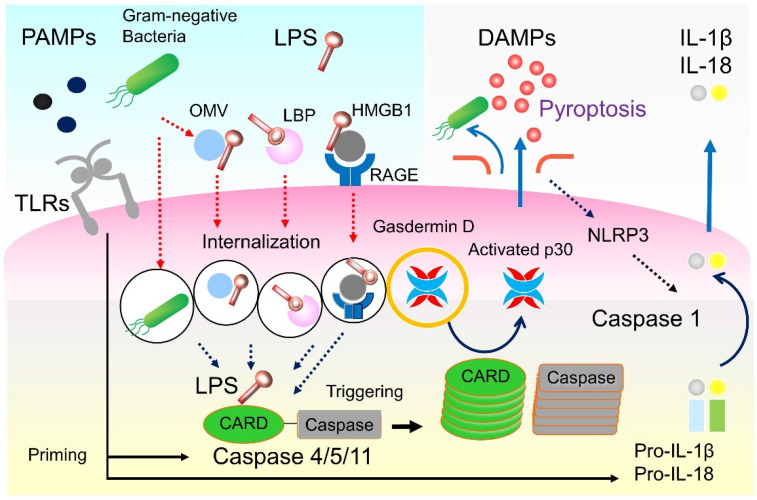
LPS internalization and pyroptosis. Hepatocyte-released HMGB1 directly binds to LPS, and the HMGB1-LPS complex is internalized via RAGE-mediated endocytosis. HMGB1 then facilitates LPS translocation from endolysosomes to the cytosol by inducing lysosomal rupture. The cytoplasmic LPS eventually activates caspase-11-dependent pyroptosis and induces lethality associated with endotoxemia. CARD, caspase activation and recruitment domains; DAMPs, damage-associated molecular patterns; HMGB1, high mobility group box-1 protein; IL, interleukin; LBP, LPS binding protein; LPS, lipopolysaccharide; NLRP3, NOD-LRR-, and pyrin domain containing protein 3; PAMPs, pathogen-associated molecular patterns; RAGE, receptor for advanced glycation end products; TLR, Toll-like receptor; OMV, outer membrane, vesicle.

Although LPS is supplied to cells when bacteria are phagocytosed or when membrane vesicles released by bacteria migrate into cells [44], there have been no new reports on the mechanism whereby free LPS is taken up into cells, except for the mechanism by which LBPs shuttle LPS into cells [45]. In 2018, Deng et al. demonstrated that the hepatocyte-released HMGB1, a representative DAMP, mediates caspase-11-dependent pyroptosis and lethality in sepsis by delivering extracellular LPS into the cytosol of macrophages and endothelial cells, where LPS activates caspase-11 [46]. In their experiment where biotin-labeled LPS was intraperitoneally administered into the peritoneal cavity of mice, the recombinant HMGB1 protein enabled LPS to induce macrophage pyroptosis, which largely depended on caspase-11. HMGB1-LPS complexes are taken up by macrophages and endothelial cells via receptors for advanced glycation end products (RAGE) [46] (Figure 2). HMGB1 interacts with LPS to mediate caspase-11-dependent pyroptosis during endotoxin shock. These findings suggest that intracellular caspase-11 and GSDMD molecules [9,38], as well as LPS and HMGB1 in the blood, are the targets of endotoxin shock treatment [10,36,47,48,49,50]. As discussed later, LPS and HMGB1 in the blood can be removed by blood purification.

## 4. Cytokines, Neutrophils, and DAMPs in Sepsis

### 4.1. Cytokines 

**Figure 3:**
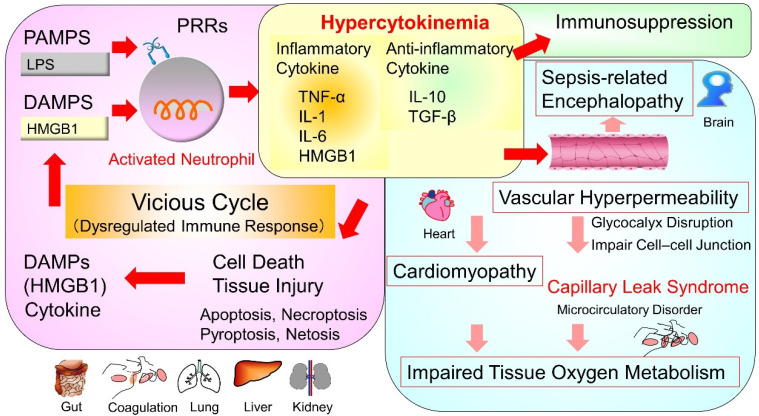
Pathophysiology of sepsis-pathological evolution from hypercytokinemia. Hypercytokinemia is widely accepted as the mainstay of the pathophysiology of sepsis. And subsequent biological reactions are thought to evolve into molecular cell-level disorders, such as cell death, microcirculatory disturbances, and mitochondrial dysfunction, as well as organ-level disorders, such as septic cardiac dysfunction and sepsis-related encephalopathy. The increased permeability of the vascular endothelium and the resulting interstitial edema and impaired tissue oxygen metabolism are of particular importance in the pathogenesis of sepsis. PAMPs, pathogen-associated molecular patterns; DAMPs, damage-associated molecular patterns; PRRs, pattern-recognition receptors; LPS, lipopolysaccharide; HMGB1, high mobility group box l protein; TNF-α, tumor necrosis factor-α; IL, interleukin; TGF-β, transforming growth factor-β.

Sepsis has now been defined as a dysregulated host response to infection, leading to life-threatening organ dysfunction, and is associated with a strong stimulation of PRRs by PAMPs and DAMPs, leading to the production of proinflammatory cytokines, such as tumor necrosis factor (TNF)-α, IL-1β, IL-6, and IL-8 [3]. The term ‘cytokine storm’ has been increasingly used in both scientific literature and public media to denote an out-of-control inflammatory response or hypercytokinemia [51]. Subsequent biological reactions are thought to progress to molecular cell-level disorders, such as cell death, microcirculatory disturbances, and mitochondrial dysfunction, as well as organ-level disorders, such as lung injury [37], sepsis-associated acute kidney injury (AKI) [52,53], septic cardiac dysfunction [54], and sepsis-related encephalopathy [55] (Figure 3).

The increased permeability of the vascular endothelium and the resulting interstitial edema and impaired tissue oxygen metabolism are of particular importance in the pathogenesis of sepsis [56]. On the surface of normal vascular endothelial cells, there are structures called glycocalyx [57]. Glycocalyx contributes to various vascular functions, such as antithrombogenicity, suppression of cellular adhesion, and selective permeability [58]. Disruption of the glycocalyx layer due to heparan sulfate degradation induced by TNF-α-dependent heparanase activation [59], and endothelial apoptosis leads to an increase in permeability to proteins and fluids, causing interstitial leakage [60]. IL-6 and HMGB1 have been shown to impair cell–cell connections by decreasing vascular endothelial cadherin and the zona occludens tight junction [61,62]. These responses contribute to local control of infection; however, systemic activation can lead to microvascular thrombosis, capillary permeability, hypotension, tissue hypoxia, and ultimately tissue damage [63] and life-threatening organ failure [64]. Experimental studies have examined possible glycocalyx-protective therapeutics [65,66] and different endothelial-specific anti-inflammatory strategies during sepsis, including blood purification techniques [67].

### 4.2. Neutrophils

Among white blood cells, neutrophils play an important role in the defense of the body by innate immunity and phagocytose-invading pathogens. However, in severe conditions under hypercytokinemia, their lifespan, which is normally short, is prolonged, resulting in persistent tissue damage induced by reactive oxygen species, proteolytic enzymes and cytokines [68,69]. Neutrophils in the peripheral blood lose their chemotaxis direction. Furthermore, the expression of CXC chemokine receptor 2 (CXCR2), which is necessary for neutrophils to migrate out of blood vessels, is decreased [70,71]. As a result, neutrophils cannot migrate to the site of infection. Vascular damage is a common feature of several highly prevalent forms of sepsis. The recruited neutrophils are detrimental rather than protective as they release toxic cargo that compromises vascular integrity or induces thrombosis [72].

Neutrophils are the first line of defense against bacterial infection, and the formation of neutrophil extracellular traps (NETs) is an important protective mechanism [73]. However, NETs can also cause harm by exposing cytotoxic histones and promoting intravascular coagulation and tissue damage in sepsis [74,75,76]. Neutrophils activated by PAMPs, DAMPs, cytokines, and other dangerous molecules are thought to be important immune cells that cause tissue damage [77]. As described above, neutrophils transition from powerful antimicrobial protectors into dangerous mediators of tissue injury and organ dysfunction [78,79]. The intimate but complex involvement of neutrophils in sepsis makes them exciting targets for therapeutic intervention. Several approaches to therapeutically target neutrophils have emerged [80], including strategies for removal from the blood [81].

### 4.3. DAMPs

Cytokine storms have generally been described as a collection of clinical manifestations resulting from an overactivated immune system. Despite the role of cytokine storm in tissue damage and multiorgan failure, a systematic understanding of its underlying molecular mechanisms is lacking [82]. Recent studies revealed a deleterious cycle between cytokine release and cell death pathways; certain cytokines, PAMPs, and DAMPs can activate inflammatory cell death, leading to further cytokine secretion [13,14,83]. Recent studies have highlighted mechanistic overlaps and extensive, multifaceted crosstalk between pyroptosis, apoptosis, and necroptosis, known as PANoptosis [84].

Cell death, which has the morphological characteristics of necrosis-releasing DAMPs, such as cell swelling, formation of pores in the cell membrane, and collapse of the cell membrane [necroptosis], is the problem associated with sepsis. Apoptosis-inducing proteins, such as TNF-α, can induce necrosis under certain conditions [85]. TNF-α-dependent necroptosis is an RIPK3-dependent cell death [86] and a programmed cell death that leads to further downstream MLKL activation and membrane lysis [87]. This cell death leads to the release of DAMPs from the cell by disruption of the cell membrane, which in turn triggers an inflammatory response [88]. Pyroptosis, such as necroptosis, is also controlled by cell death accompanied by membrane lysis. This membrane lysis causes cytotoxic efflux of not only activated IL-1β and IL-18, which are cleaved by caspases, but also DAMPs, such as HMGB1 and mitochondrial deoxyribonucleic acid, from dead cells. DAMPs released from dead or injured cells are recognized by PRRs on the surface of macrophages or inside cells, and amplify the sustained response of further cytokine secretion, leading to physiological disorders, such as cytokine storms [89]. Thus, once the “negative spiral” or “damaged chain reaction” begins to turn, even if the pathogen is controlled, the inflammation metastasizes and amplifies, which is the mechanism of the “uncontrolled immune response” [90].

## 5. Extracorporeal Blood Purification Techniques

### 5.1. Rationale

**Figure 4:**
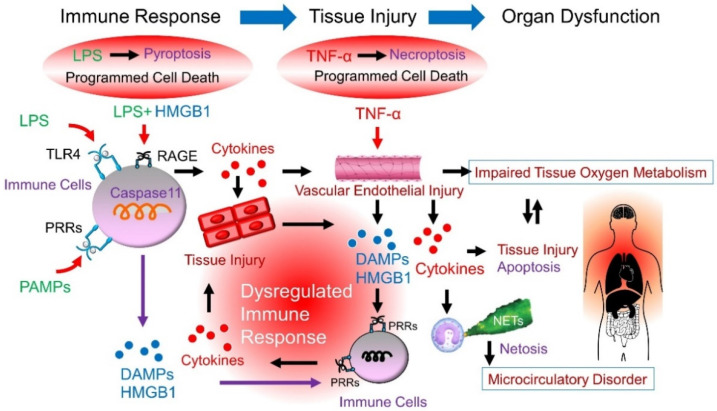
Target molecules for blood purification in sepsis. Infection stimulates immune cells with PAMPs and LPS, resulting in the production of cytokines, such as TNF-α. Cytokines induce vascular endothelial damage and impair tissue oxygen metabolism. In addition, TNF-α induces necroptosis in the cells. Injured cells release DAMPs, such as HMGB1. However, LPS is taken up by cells and induces pyroptosis, resulting in the release of DAMPs. The released/produced DAMPs are recognized by the receptors (PRRs) of immune cells and secondarily produce inflammatory cytokines, triggering a persistent vicious cycle (i.e., uncontrolled host response). Considering the pathogenesis of sepsis, the targets of blood purification are considered to be LPS and immune cells, cytokines, and DAMPs. LPS, lipopolysaccharide; TNF-α, tumor necrosis factor-α; HMGB1, high-mobility group box l protein; TLR4, toll-like receptor 4; PRRs, pattern recognition receptors; PAMPs, pathogen-associated molecular patterns; DAMPs, damage-associated molecular patterns; NETs, neutrophil extracellular traps; PRRs, pattern-recognition receptors; RAGE, receptor for advanced glycation end products.

Sepsis is one of the leading causes of death worldwide; however, there are no therapies available besides intensive care unit (ICU) treatment. Initial attempts at drug development mainly focused on controlling inflammation, but without any tangible outcomes. Recently, uncontrolled cytokine storms have been thought to be driven by the interplay between inflammatory signaling and inflammatory cell death [91]. The rationale behind the blood purification approach is to achieve “immune homeostasis,” which reduces a remarkable increase in inflammatory mediators, including LPS, cytokines, and DAMPS [92,93,94,95,96]. These mediators have been shown to cause direct vascular endothelial damage [60] or be filtered in the glomeruli, and then be exposed to TLRs present on the proximal tubular epithelial cell surface, leading to sepsis-associated AKI [52,53]. This may be significant for lowering the concentration of mediators in the blood (Figure 4).

Early studies suggested that high-volume hemofiltration [97,98] or high cut-off dialysis [99], using the principle of filtration, could reduce cytokine levels. Further, very low-quality evidence has demonstrated that the use of blood purification may reduce mortality in sepsis or septic shock [100]. As will be discussed later, theoretically, the filtration principle is limited to the filtrate flow rate; however, the adsorption principle can increase mediator clearance (CL) up to the blood flow rate [101].

Adsorption is more efficient for the removal of mediators with large molecular weights, such as cytokines [102,103]. Cytokine-adsorbing hemofilters are easy to use in the ICU because they can achieve both mediator removal and renal support therapy [104,105]. We focused on blood purification devices that remove mediators via the adsorption mechanism in sepsis. Additionally, as a future technology, we present the immune-modulation technology currently under investigation.

### 5.2. Cytokine-Adsorbing Hemofilter

**Figure 5:**
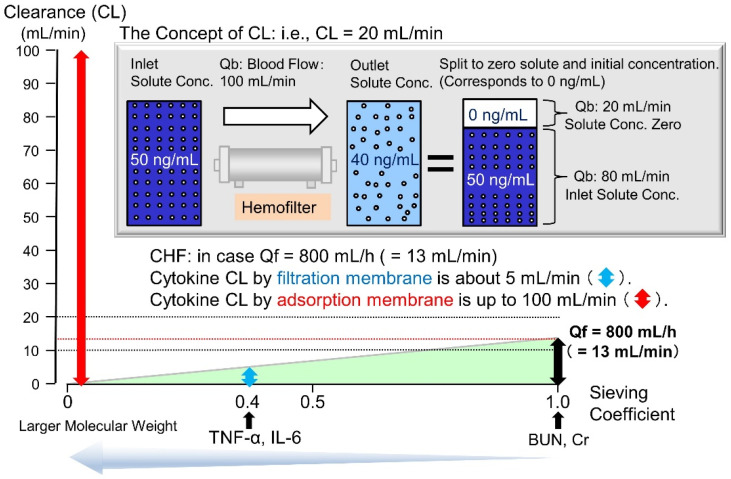
The concept of clearance and the limits of filtration during blood purification. Clearance (CL) is used as an indicator of the ability of a hemofilter to remove substances. It is expressed as the blood flow rate (mL/min) corresponding to the amount of blood flowing into the hemofilter that has been completely removed (i.e., the concentration is zero). When calculating CL, the concentration after passing through the hemofilter can be divided into the original inlet concentration and the flow rate at which the concentration is completely zero. The CL was used as an index of the removal capacity of the hemofilters. CL was calculated from the cytokine concentration obtained using the aforementioned method. Blood CL = (CBi-CBo)/CBi × (QB-QF/60) + QF/60 (CBi, concentration before filter; CBo, concentration after filter; QB, blood flow rate [mL/min]; QF, filtrate flow rate [mL/h]). In the case of hemofiltration at a filtrate flow rate of 800 mL/h (13 mL/min, the CL of Cr (sieving coefficient = 1.0) was 13 mL/min. Using the same calculation, the filtration CL of the cytokine (molecular weight of 20,000, sieve factor of approximately 0.4) was approximately 5 mL/min (=13 mL/min × 0.4). However, for adsorption, it is theoretically possible to set the outlet concentration to zero, which would result in a maximum adsorption CL of 100 mL/min (=blood flow rate). TNF-α, tumor necrosis factor-α; IL-6 interleukin-6; BUN, blood urea nitrogen; Cr, creatinine.

Polymethylmethacrylate (PMMA) and acrylonitrile/sodium methallylsulfonate (AN69ST) hemofilters, also known as cytokine-adsorbing hemofilters, are known to adsorb inflammatory mediators [21,104,105,106,107,108]. Further, continuous hemo(dia)filtration (CH(D)F) has a history of being performed not only to replace renal function, but also to prevent or treat organ damage through the removal of inflammatory mediators in Japan [109]. However, prior to our in vitro study [110,111,112], little was known about the quantity of cytokines adsorbed on the cytokine adsorption membrane and the mechanism of adsorption. In the principle of membrane filtration, only substances smaller than the membrane pores can be removed. Normally, the pores of membranes are designed such that albumin (molecular weight 66 kDa) cannot pass through membrane, and cytokines (molecular weight 20–30 kDa) can only be slightly removed (Figure 5).

**Figure 6:**
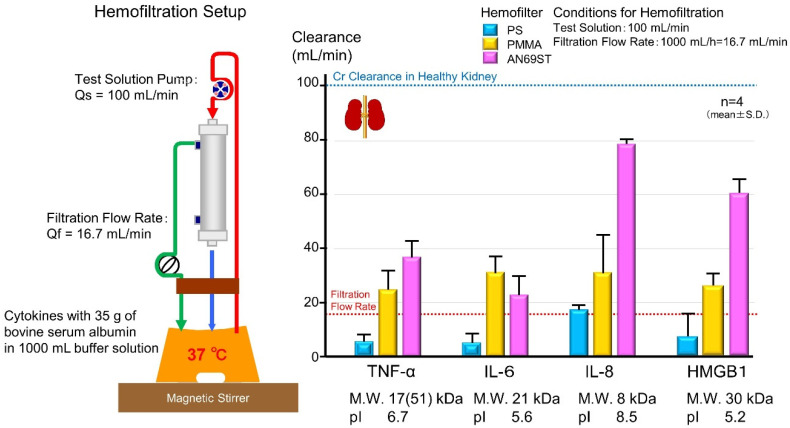
Cytokine CL during hemofiltration. The in vitro experimental setup was comprised of a solution reservoir. The investigated hollow-fiber hemofilters were AN69ST, PMMA, and a polysulfone (PS) hemofilter. A hemofilter and the extracorporeal circuit were primed with 1 L of heparinized saline solution. The test solution was prepared by dissolving cytokines with different isoelectric points (pI) and molecular weights (kDa), including TNF-α (pI 6.5; 17 kDa), IL-6 (pI 5.3; 21 kDa), IL-8 (pI 8.5; 8 kDa), and HMGB1(pI 5.2; 30 kDa) with 35 g of bovine serum albumin in 1000 mL of buffer solution (pH 7.2). The test solution was pumped from the reservoir solution to the hemofilter at a solution flow rate (Qs) of 100 mL/min, and then returned to the same reservoir. The ultrafiltrate was pumped at a filtrate flow rate (Qf) of 1000 mL/h for 16.7 mL/min and returned to the reservoir in a closed-loop circulation system. The test-solution CL of a cytokine represents a direct index for evaluating the adsorption ability of a membrane. Both PMMA and AN69ST hemofilters showed high CL above the Qf (16.7 mL/min), which was the theoretical limit of the filtration mechanism. In addition, the CLs of the cytokine adsorbing membrane are large as the CL of Cr in healthy kidney is approximately 100 mL/min. This figure is summarized from Yumoto 2011 [110], and Moriyama 2020 [111]. M.W., molecular weight; pI, isoelectric point; Cr, creatinine; TNF-α, tumor necrosis factor-α; IL-6, interleukin-6; IL-8, interleukin-8; HMGB1, high mobility group box 1; Cr, creatinine; PS, polysulfone; PMMA, polymethylmethacrylate; AN69ST, acrylonitrile/sodium methallyl sulfonate.

In an in vitro experimental system, the researchers showed that the CL of HMGB1 [110], TNF-α, IL-6, and IL-8 [111] by cytokine adsorbing membranes, such as PMMA and AN69ST, exceeded the upper limit of filtration CL (filtrate flow rate) (Figure 6). Additionally, HMGB1 with a molecular weight of approximately 30,000 did not saturate the AN69ST membrane. When HMGB1 was repeatedly added seven times during hemofiltration, a rapid decrease in circulating HMGB1 was observed after every addition, with no signs of saturation [112]. The AN69ST membrane with adsorbed HMGB1 was observed using immunoelectron microscopy. Even when a high concentration of HMGB1 was adsorbed, HMGB1 was observed on the surface of the membrane and in some bulk layers. Thus, the membrane surface was identified to be too large for saturation to occur [112]. Furthermore, changing the hydrogen ion concentration of the test solution at each step in cytokine adsorption experiments revealed that ionic binding was the mechanism employed for cytokine adsorption by the AN69ST membrane (negatively charged) [111]. AN69ST-CHDF was evaluated in a prospective, multicenter clinical study in Japan. The CLs of all measured cytokines (TNF-α, IL-1β, IL-6, IL-8, IL-10, and HMGB1) were found to exceed the filtration flow rate, and IL-8 (positively charged) showed the highest CL, similar to results of our in vitro experiment [105].

We also confirmed that the pore size of PMMA membranes was larger than that of AN69ST in experiments using myoglobin, which had no adsorption properties [113], suggesting that even IL-6 with a large molecular weight can be trapped on the PMMA membrane. The mechanism of cytokine adsorption by PMMA seems to involve hydrophobic binding, but this is not fully understood. Additionally, we confirmed that the adsorption capacity of cytokine-adsorbing hemofilters is sufficient for small amounts of substances, such as cytokines, but has no effect on antimicrobial agents, which are millions of times higher than the blood concentration of cytokines, because they exceed the saturation amount [114].

As already mentioned, AN69ST adsorbs cytokines with a large isoelectric point, such as IL-8, while PMMA can adequately adsorb cytokines with a molecular size of approximately 20 kDa, such as IL-6 [111]. In particular, when selecting membranes for cytokine removal, molecular weight and isoelectric point are helpful. Since 2014, AN69ST membranes have been covered by health insurance in Japan for patients with sepsis and septic shock without renal dysfunction [105,115]. A retrospective observational study was conducted after the launch of this product, which suggested that CH(D)F with an AN69ST hemofilter might be associated with better mortality or in-hospital outcomes [116,117]. However, there is currently no high-level evidence of the clinical efficacy of adsorption membranes.

### 5.3. LPS Absorptive Column

Endotoxin removal therapy with polymyxin B immobilized fiber column (PMX) has been clinically applied to patients with septic shock since 1994 [118]. Direct hemoperfusion with PMX (PMX-DHP) has caused improvement in organ dysfunction and a survival benefit in small studies [119], including a small randomized trial [120], while larger trials failed to confirm these findings [121,122]. The Evaluating the Use of Polymyxin B Hemoperfusion in a Randomized Controlled Trial of Adults Treated for Endotoxemia and Septic shock (EUPHRATES) trial was a double-blinded randomized controlled trial conducted in North America [122]. This trial enrolled 450 adult septic shock patients with an EAA level ≥ 0.6 and multiple organ dysfunction score (MODS) > 9. PMX caused improvements in the mean arterial pressure and ventilator-free days. However, the mortality rate at 28 days was not significantly different between the groups. According to Dellinger et al., the relative timing, dose, and 2 h duration of PMX-DHP treatment may have been insufficient to reduce endotoxin levels and mortality [122].

In response to this, we examined whether the LPS adsorption capacity was insufficient [123]. In our experiment, LPS was continuously infused into the reservoir with bovine serum to increase the LPS concentration over time. A perfusion test with PMX-01R (small column) or with blood tubing alone as a sham control was performed. This result indicated that the adsorption capacity of PMX-01R did not saturate after 24 h. We found that only a percentage of the PMX-20R column adsorption capacity would be used within 2 h in an adult sepsis patient with an endotoxin concentration of 100 pg/mL (although 2-figure pg/mL levels or less are generally observed) [123]. If an equilibrium in adsorption does not occur, a longer duration of PMX-DHP for the right patient subset may be another strategy to determine the efficacy of PMX-DHP in patients with septic shock [43,124].

The oXiris^®^ hemofilter was developed to enhance the adsorptive properties of a previously well-studied AN69 surface-treated membrane [106]. It offers combined cytokine and endotoxin removal properties and renal support [92,125]. Despite compelling preclinical data, suitably powered randomized controlled trials with appropriate patients have not been conducted.

### 5.4. Cytokine Adsorbing Column

The CytoSorb^®^ technology (CytoSorbents, Monmouth Junction, NJ, USA) uses a hemoperfusion cartridge to absorb cytokines [126]. In vitro studies have demonstrated the optimal capacity for removing broad-spectrum cytokines with removal rates of >90% at 120 min [125]. However, there have been reports that IL-6 did not decrease in clinical cases [127], and the evidence supporting its favorable outcomes on hemodynamic parameters and blood lactate levels has been limited to a case series [128]. Despite such promising experimental results, only a randomized controlled trial has evaluated the efficacy of CytoSorb^®^ in critically ill patients with sepsis. For the 100 subjects enrolled and randomized to receive CytoSorb^®^ or no hemoperfusion, no differences in IL-6 concentration (primary endpoint) or 60-day-mortality (secondary endpoint) were observed between the treatment and control groups [129]. Cytokine adsorption capacity should be assessed by CL; however, there has been no extensive evidence of cytokine CL in vivo.

### 5.5. Immune-Modulation Therapy

Excessive activation of neutrophils plays important roles and leads to organ dysfunction [130]. Neutrophils, which contribute to tissue damage during sepsis, may also be targeted for removal [77,131,132]. Although blood purification for the adsorption of cytokines has been studied, there are no therapeutic measures for activated neutrophils. In a prior study, we developed an immune-modulating blood purification system (IMBPS) that regulates the level of immune reactions to modulate neutrophil function. This blood purification system of the “DECOY” organ system relocates the massive systemic inflammation in true organs in a body to the “IMBPS decoy organ.” Accordingly, the neutrophil activity in the blood is expected to be weakened, and as a result, organ damage could be prevented [133].

**Figure 7:**
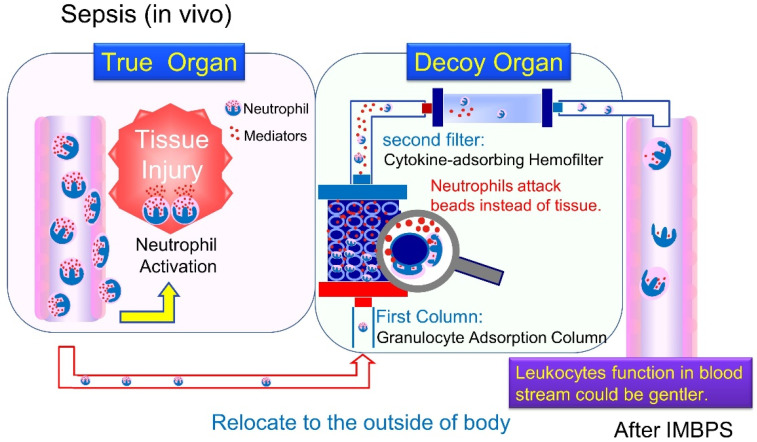
IMBPS: extracorporeal “DECOY” organ. IMBPS consists of a granulocyte adsorbing column (Adacolumn^®^) and a cytokine-adsorbing hemofilter (AN69ST hemofilter). First, leukocytes are induced into IMBPS and attack the beads in the first column. Adacolumn^®^ is an “extracorporeal decoy organ” that allows granulocytes to attack themselves instead of “true organ in a body”. The AN69ST hemofilter remove mediators that may be produced by activated neutrophils. By using this column combination system, we expect leukocytes that function in the blood stream to be gentler, preventing organ dysfunction. IMBPS, immune-modulating blood purification system.

We designed Adacolumn^®^ as a decoy upstream and an AN69ST hemofilter downstream to remove mediators produced by activated neutrophils (Figure 7). Adacolumn^®^ is composed of cellulose acetate beads 2 mm in diameter to adsorb granulocytes and monocytes [134,135]. Ulcerative colitis, Crohn’s disease, and pustular psoriasis are covered by the National Health Insurance in Japan [136]. AN69ST is highly capable of capturing cytokines [105,111]. For the ex vivo experiment with IMBPS, fresh porcine blood was circulated for 6 h. In this study, Adacolumn^®^ was found to mainly adsorb granulocytes and monocytes, but not lymphocytes. The phagocytic activity level of granulocytes decreased while adhesiveness increased; however, the number of CD11b-positive cells markedly decreased in the current system. Elevated cytokine levels (IL-1β, IL-8, and IL-10) at the outlet of Adacolumn^®^ (decoy upstream) were significantly lower than at the outlet of the AN69 hemofilter (second filter) due to cytokine adsorption [133]. This system is currently undergoing clinical studies in Japan (JapicCTI-205279).

Early attempts at drug development mainly focused on controlling inflammation, but without any tangible outcomes. Knowledge of the mechanisms involved in the pathogenesis of specific pathogens is critical for the development of novel blood purification therapies. The focus may have been shifting to understanding deleterious immune cells to develop therapy [137]. However, this hypothesis remains to be confirmed.

## 6. Perspectives and Conclusions

Remarkable progress in basic science, together with numerous clinical studies, has increased our knowledge of sepsis. Based on the recent pathophysiology of sepsis, a therapeutic approach to LPS, cytokines and DAMPs has become more attractive. Numerous mediators are involved in the immune response; however, targeting a single mediator may not be sufficient to block the complex inflammatory response in sepsis, at least during the clinical phase. Thus, over time, the blood purification concept has evolved to focus on the non-specific removal of a broad spectrum of inflammatory mediators, which can also include microbial toxins [52,94].

Adsorption is promising for the removal of mediators with large molecular weights, such as cytokines. Although adsorbents are often designed as DHP columns, CH(D)F is easier to use in the ICU, where both cytokine removal and renal support therapy can be achieved. As CH(D)F is internationally called “renal (kidney) replacement therapy” and has been applied for AKI outside of Japan [138], there has been little investigation of mediator removal for sepsis. The data presented in this review suggest the possibility of cytokine-adsorbing hemofilters or cytokine-adsorbing hemofilters with novel immune-modulation columns to treat sepsis. Despite the heterogeneity of sepsis, over the past three decades, sepsis trials have focused on a one-size-fits-all approach to treatment. Future studies should start by identifying the right patient subset at the right time and deciding on at an optimal duration or well-defined conditions to definitely evaluate the efficacy of sorbent devices [139,140].

## Data Availability

Not applicable.
